# Protecting historically marginalized groups or targeted marketing? A computational analysis of individuals engaging with public and protected cigar-branded tweets

**DOI:** 10.1016/j.drugalcdep.2024.112516

**Published:** 2024-11-29

**Authors:** Jiaxi Wu, Lynsie R. Ranker, Juan Manuel Origgi, Jianqi Ma, Deyan Hao, Emelia J. Benjamin, Jennifer Cornacchione Ross, Ziming Xuan, Derry Wijaya, Jessica L. Fetterman, Traci Hong

**Affiliations:** a Annenberg School for Communication, University of Pennsylvania, Philadelphia, PA, USA; b Department of Community Health Sciences, Boston University School of Public Health, Boston, MA, USA; c Department of Computer Science, Boston University, Boston, MA, USA; d Section of Cardiovascular Medicine, Department of Medicine, Boston Medical Center and Boston University Chobanian & Avedisian School of Medicine, Boston, MA, USA; e Department of Epidemiology, Boston University School of Public Health, Boston, MA, USA; f Department of Health Law, Policy & Management, Boston University School of Public Health, Boston, MA, USA; g Department of Data Science, Monash University, Bumi Serpong Damai, Banten, Indonesia; h Evans Department of Medicine and Whitaker Cardiovascular Institute, Boston University Chobanian & Avedisian School of Medicine, Boston, MA, USA; i College of Communication, Boston University, Boston, MA, USA

**Keywords:** Social media, Twitter, Tobacco marketing, Flavored cigars, Machine learning

## Abstract

**Introduction:**

Swisher Sweets, a leading brand of little cigars and cigarillos in the United States, switched its Twitter account to protected status, limiting access to its tweets. This study examines how the protected status of Swisher Sweets tweets influences post engagement, aiming to inform regulatory strategies for branded tobacco promotions on social media.

**Method:**

Using natural language processing, we predicted the demographics of individuals replying to Swisher Sweets’ public and protected tweets. Engagement with public versus protected tweets was compared using a Mann-Whitney U test, and a mixed-effects logistic regression assessed the likelihood of different demographics replying to each tweet type. Chi-square analyses examined word frequencies related to any flavor, concept flavors, and characterizing flavor in replies to public and protected tweets.

**Results:**

Overall, 16 % of individuals replying to Swisher Sweets’ tweets were predicted to be under 21, and 65 % were Black. No significant difference was found in average reply counts to public versus protected tweets (p = .78). Black individuals were 2.61 times more likely than White individuals to engage with protected tweets after the status change (OR = 2.61, 95 % CI [1.36, 5.06], p = .004). Replies to protected tweets contained more words related to any flavor (adjusted p < .001) and concept flavors (adjusted p < .001) compared to public tweet replies.

**Conclusions:**

Our study suggests that the protected status on Twitter was ineffective in preventing underage engagement with Swisher Sweets’ branded tweets and may facilitate targeted marketing among Black individuals.

**Implications:**

More stringent age verification procedures and promotional regulatory measures are needed to prevent targeted tobacco brand marketing on social media.

## 1. Introduction

In 2023, 1.3 million US youth reported ever using cigar products (Birdsey et al., 2023). Compared to premium large cigars, little cigars and cigarillos (LCCs) are particularly popular among youth (Hair et al., 2021). The use of LCCs among youth is, in part, driven by the availability of different flavors that are prohibited in cigarettes (Cullen et al., 2019; Dai and Hao, 2019).

LCC companies use targeted marketing tactics to promote cigar products to communities of color (Navarro et al., 2020; Richardson et al., 2014; Kong et al., 2020). LCC products are increasingly marketed on social media, with a study revealing that 19 out of 24 leading cigar brands maintain accounts on at least one platform (O’Brien et al., 2020). Evidence suggests that exposure to and engagement with tobacco marketing on social media is linked to tobacco use among youth and young adults (Clendennen et al., 2020; Ranker et al., 2024).

Swisher Sweets is a leading LCC brand in the US, accounting for more than 25 % of cigar sales in convenience stores between 2009 and 2020 in the US (Delnevo et al., 2021). The official Twitter account, @SwisherSweets was created in July 2011, accumulating 23 K followers and sharing 4 K+ tweets as of September 2023. The Twitter account is also linked on the Swisher Sweets website, suggesting direct brand management. While most tobacco brands stay public to engage audiences (O’Brien et al., 2020; Kong et al., 2023; Vassey et al., 2023), Swisher Sweets made its account protected around August 23, 2019. In protected status, only followers who have requested and been approved by the account can view and reply to tweets, while retweeting is disabled for all Twitter users. Profile details, such as bios, profile photos, and follower/following lists, remain visible and searchable to all . The reason for this switch is unclear. While no research currently documents the prevalence of protected social media accounts among LCC brands on Twitter, Swisher Sweets’ protected status may indicate an industry trend toward using privacy settings to manage engagement and regulatory visibility.

Evidence increasingly indicates that exposure to and engagement with tobacco marketing on social media can significantly influence tobacco use initiation and continuation among young people (Ranker et al., 2024; Richardson et al., 2024). Social media algorithms prioritize popular content based on engagement, creating a feedback loop that repeatedly exposes young audiences to similar content, heightening their susceptibility to tobacco use (Soneji et al., 2018). This cyclical exposure is especially concerning as young individuals are highly receptive to social and environmental influences, making them more likely to initiating and sustaining tobacco use (Rutherford et al., 2023).

This study examines engagement in tweet replies before and after the Swisher Sweets account status change to reveal the broader implications of such brand activities.

## 2. Methods

### 2.1. Study design and data collection

Because protected tweets cannot be retweeted, we inferred that Swisher Sweets switched its Twitter account to protected status around August 23, 2019, as no tweets from Swisher Sweets have been retweeted by any Twitter users since that date. Each reply to the Swisher Sweets account contains the handle @SwisherSweets and is accessible through Brandwatch as long as the Twitter user replying has a public account, regardless of whether the reply is directed at the public or protected Swisher Sweets account. We used the Brandwatch Twitter firehose dataset to collect all publicly available replies to the Swisher Sweets account from April 13, 2018 to December 30, 2020. This time frame spans around the same days before (n = 496) and after (n = 496) Swisher Sweets changed its account to protected, representing a total of 992 days. Overall, we collected 1571 replies to Swisher Sweets’ tweets. In total, the public reply dataset comprised 929 replies from 576 distinct Twitter accounts, while the protected reply dataset had 642 replies from 437 unique Twitter accounts. Additionally, 67 Twitter accounts responded to both public and protected tweets from Swisher Sweets. The study protocol was reviewed by the IRB of the authors’ research institution and determined to not pertain to human subjects’ research due to the use of publicly available data.

### 2.2. Demographic prediction of twitter users replying to the Swisher Sweets Twitter account

Using the Twitter public API, we scraped the most recent 100 tweets and profile data for 576 public and 437 protected users, capturing their Twitter handles, screen names, bios, and profile images. To enhance model accuracy, demographic prediction was restricted to accounts with at least 50 tweets. Due to account suspensions and privacy settings, we could predict demographics for 435 (76 %) public and 369 (84 %) protected users. Model accuracy was evaluated with a pre-labeled dataset of 3000 Twitter users with known sex, age, and race.

We utilized the M3 inference model to predict users’ sex (female vs. male) by analyzing their Twitter bios, handles, usernames, and profile images (when available) (Wang et al., 2019). This open-source deep learning model, pre-trained on over five million Tweets, categorized users with a probability score of ≥ 0.5 as male and < 0.5 as female. Before applying M3, we validated it on a dataset of 3000 Twitter users who disclosed their sex, achieving 91.5 % accuracy.

For age prediction, we developed a logistic regression model and employed a term frequency-inverse document frequency vectorizer. A pre-labeled dataset of 3000 Twitter users with profile disclosures of age was used for the model development. Accounts with a ≥ 50 % probability of being ≥ 21 years (tobacco purchasing age post-Tobacco 21) were categorized as such, while others were considered underage. To evaluate the model performance, we employed K-Fold validation, wherein the dataset was divided into five equally sized and smaller subsets. Our model achieved 70 % accuracy in predicting users’ ages as < 21 or ≥ 21 years.

We developed a custom model to predict Twitter users’ race. The model has two components: 1) a feature extraction using unigrams, bigrams, and up to 5000 features for TF-IDF, and 2) a logistic regression classifier to predict race among Black, White, and Latino-Asian. The classifier achieved 71 % accuracy across the three racial categories.

### 2.3. Flavor-related words in comments

We obtained a list of flavors on Swisher Sweets’ official website. Then, we categorized all of the flavors into characterizing flavors and concept flavors (Delnevo et al., 2021). Concept flavors included diamonds, green, silver, wild rush, black, aromatic, smooth, summer twist, and tropical fusion. Characterizing flavors consisted of berry, blueberry, chocolate, grape, mango, peach, strawberry, honey, cognac, wine, and cherry. We performed non-case-sensitive exact string matching for all flavors, descriptive flavors, and conceptual flavor to identify the presence of flavor-related words in each comment.

### 2.4. Statistical analyses

Due to the non-normal distribution of reply counts, we used a two-sided Mann-Whitney *U* test to compare average replies between public and protected Swisher Sweets tweets. To address non-independence from 67 overlapping users, we employed mixed-effects logistic regression, incorporating demographics (age, sex, race). Chi-square analyses assessed the presence of flavor-related words in comments for public versus protected tweets, adjusted for multiple testing using the Bonferroni correction.

## 3. Results

### 3.1. Replies to public and protected Swisher Sweets’ tweets

The 929 replies from 576 unique Twitter users in the public dataset were associated with 253 Swisher Sweets public tweets, while the 642 replies from 437 unique Twitter users in the protected dataset were linked to 133 protected tweets. In a Mann-Whitney *U* test, no statistical difference was found between the average number of replies to Swisher Sweets’ public tweets (M = 3.67, SD = 5.14) versus protected tweets (M = 4.83, SD = 8.30) (p = .78).

In addition, 18.5 % of replies to public Swisher Sweets tweets and 32.7 % of replies to protected tweets contained flavor-related words. Chi-square analyses showed that protected tweets had more flavor-related words overall (adjusted p < .001) and more concept flavors (adjusted p < .001) compared to public tweets. No significant difference was found in characterizing flavor-related words between the two types of tweets (adjusted p = .11) (Table 1).

### 3.2. Demographics of individuals replying to Swisher Sweets’ public and protected tweets

Overall, about 16 % of individuals who replied to Swisher Sweets’ public and protected tweets were predicted to be under 21. Specifically, 70 (16 %) of those who replied to public tweets and 62 (17 %) of those who replied to protected tweets were underage. Of the 62 underage users replying to protected tweets, 53 had not engaged with Swisher Sweets’ tweets prior to the status change. Additionally, 65 % of all repliers were predicted to be Black. Specifically, 60 % of replies to public tweets and 69 % of replies to protected tweets were from Black individuals. Among users under 21, 73 % of replies to public tweets and 81 % to protected tweets were from Black individuals.

In mixed-effects logistic regression models, underage users had similar odds of replying to protected tweets when compared to users ≥ 21-years-old (OR = 0.88, 95 % CI [0.46, 1.68], *p*=.70). Compared to females, males also had similar odds of replying to protected Swisher Sweets tweets (OR = 0.86, 95 % CI [0.48, 1.53], *p*=.60) after the status change. Compared to White individuals, Black individuals are 2.61 times more likely to reply to protected Swisher Sweets posts (OR = 2.61, 95 % CI [1.36, 5.06], *p*=.004) (Table 2).

## 4. Discussion

This study examined the demographics of individuals replying to Swisher Sweets’ tweets before and after the account’s protected setting. The increased likelihood of Black individuals engaging with protected Swisher Sweets tweets, compared to White individuals, may reflect the brand’s targeted marketing efforts (Navarro et al., 2020; Kong et al., 2020). Additionally, protected tweets attracted 53 new underage users who had not engaged with public tweets. These findings suggest that the protected status is inadequate in preventing underage interactions with tobacco-branded content on social media.

Replies to protected Swisher Sweets tweets mentioned more flavor- and concept-flavor-related words compared to replies to public Swisher Sweets tweets. This result may reflect a broader trend and marketing strategy. Between 2009 and 2020, sales of concept-flavored cigars in US convenience stores increased from 2 % to 21 % (Delnevo et al., 2021). Cigars labeled with a concept flavor could circumvent state and local flavored tobacco bans that prohibit the use of characterizing flavors (Gammon et al., 2019). Our study lends support for the prohibition of flavors, including concept flavors, in cigar products to decrease the appeal of cigars among youth. Another possible explanation is that we sourced the flavor list from Swisher Sweets’ website in January 2022, which may have excluded seasonal flavors available previously. Future research should develop more comprehensive lists of Swisher Sweets or LCCs’ concept and characterizing flavors to better investigate the emergence and promotion of concept flavors.

This study has several limitations. First, the natural language processing methods used for demographic prediction may introduce bias, potentially affecting the accuracy of the findings. Additionally, focusing on a single brand and platform limits the applicability of the results to other brands and social media environments. By analyzing only tweet replies, we may not fully capture the brand’s influence, such as influencer promotions. Lastly, although “underage” is defined as under 21 according to the Tobacco 21 policy (effective December 2019), our data collection from April 2018 to December 2020 includes replies from users aged 18–20 who were legally permitted to purchase tobacco before this change.

The 1998 Master Settlement Agreement and the 2009 Family Smoking Prevention and Tobacco Control Act did not address social media advertising, allowing the tobacco industry to operate in an unregulated digital sphere with wide reach. Our findings reflect a broader trend in tobacco marketing, where digital platforms are strategically used to target younger audiences, creating new challenges for traditional public health policies and regulations. The use of protected social media accounts by tobacco brands reveals a notable gap in current regulatory frameworks, as existing policies often overlook digital engagement tactics that evade age restrictions and obscure content visibility. This study emphasizes the urgent need for updated tobacco control strategies that address the intricacies of digital marketing. Enhancing regulatory oversight, enforcing stricter age-verification measures, and developing social media-specific guidelines could be essential in limiting youth exposure to tobacco promotions online.

## Figures and Tables

**Table 1:** Flavor-related words in replies to public and protected Swisher Sweets tweets.

Swisher Sweets Replies Contain words related to:	Replies to Public Swisher Sweets Tweets* (N = 929)	Replies to Protected Swisher Sweets Tweets (N = 642)	Adjusted *p value*	OR (95 % CI)
**Any flavors**	172 (18.5 %)	210 (32.7 %)	**< .001**	2.14 (1.69, 2.70)
**Characterizing flavors**	89 (9.6 %)	83 (12.9 %)	.11	1.40 (1.02, 1.93)
**Concept flavors**	87 (9.4 %)	132 (20.6 %)	**< .001**	2.50 (1.87, 3.35)

* Reference group. OR = odds ratio for the presence of words; CI = confidence interval. *p* values were adjusted with Bonferroni correction methods.

**Table 2:** Predicting odds of replying to protected Swisher Sweets tweets with predicted demographic features.

Variable	Coefficient	Standard error	p value	OR [95 % CI]
Intercept	− 0.92	0.37	**0.01**	0.40 [0.19 – 0.82]
Age < 21	− 0.13	− .33	0.70	0.88 [0.46 – 1.68]
Black	0.96	0.33	**0.004**	2.61 [1.36 – 5.06]
Other races	0.32	0.40	0.42	1.38 [0.63 – 3.04]
Males	− 0.15	0.30	0.60	0.86 [0.48 – 1.53]

The reference group for age comparison was age ≥ 21-year-old; the reference group for race comparison was White; the reference group for gender comparison was females.

## Data Availability

The data underlying this article will be shared on reasonable request to the corresponding author.
